# Supplementary figures and images for: Brain age gap reduction following exercise mirrors clinical improvements in schizophrenia spectrum disorders
Source: Neuroimage Clin. 2025 Sep 19;48:103881. doi: 10.1016/j.nicl.2025.103881 (PMC12538700; doi:10.1016/j.nicl.2025.103881)

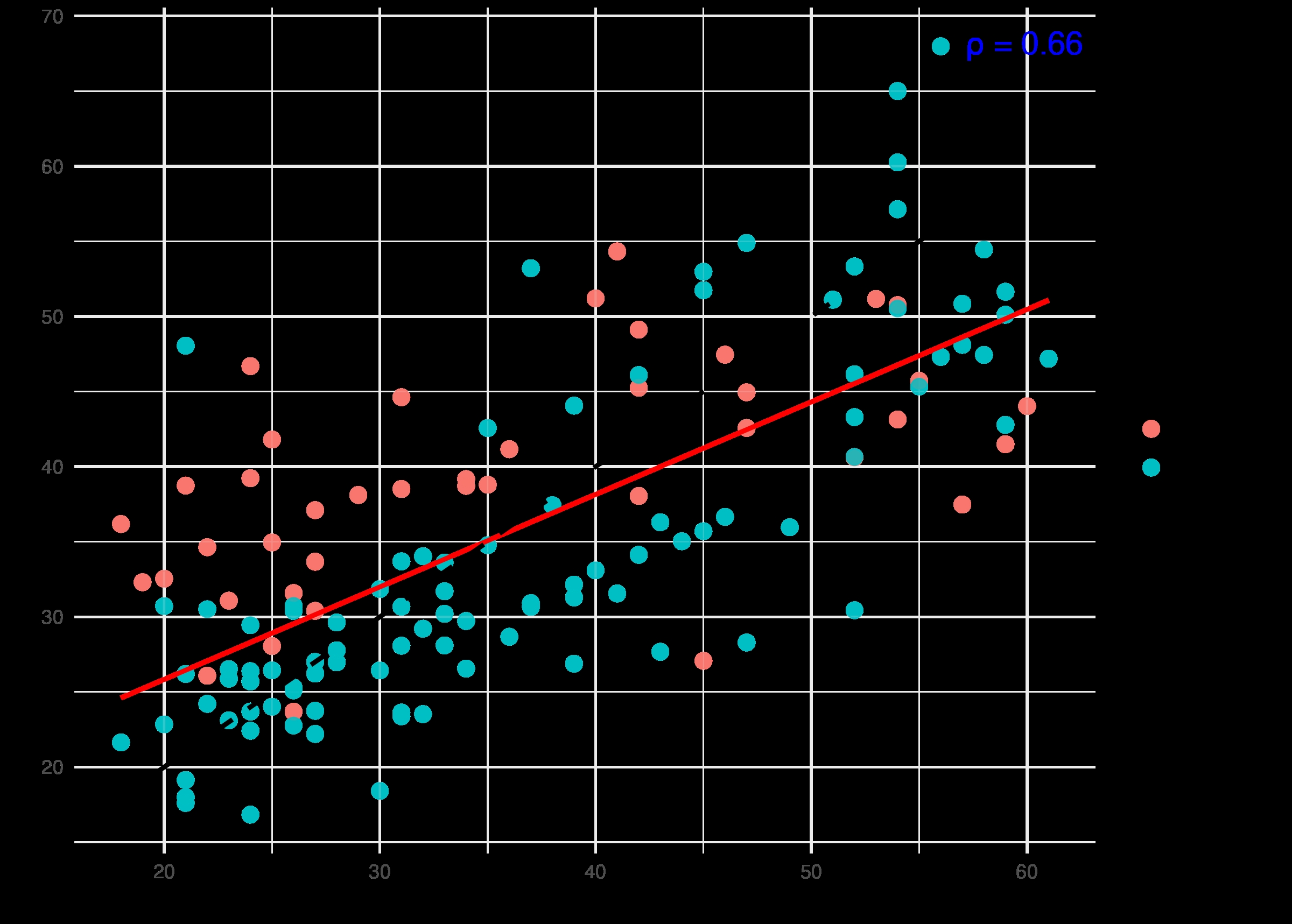

Supplement: Supplementary Figure 2 — Scatter Plot of Age vs. Brain-Predicted Age. Note. The dashed line represents the identity line (y = x), and the red line represents the regression line [file mmc2.jpg]

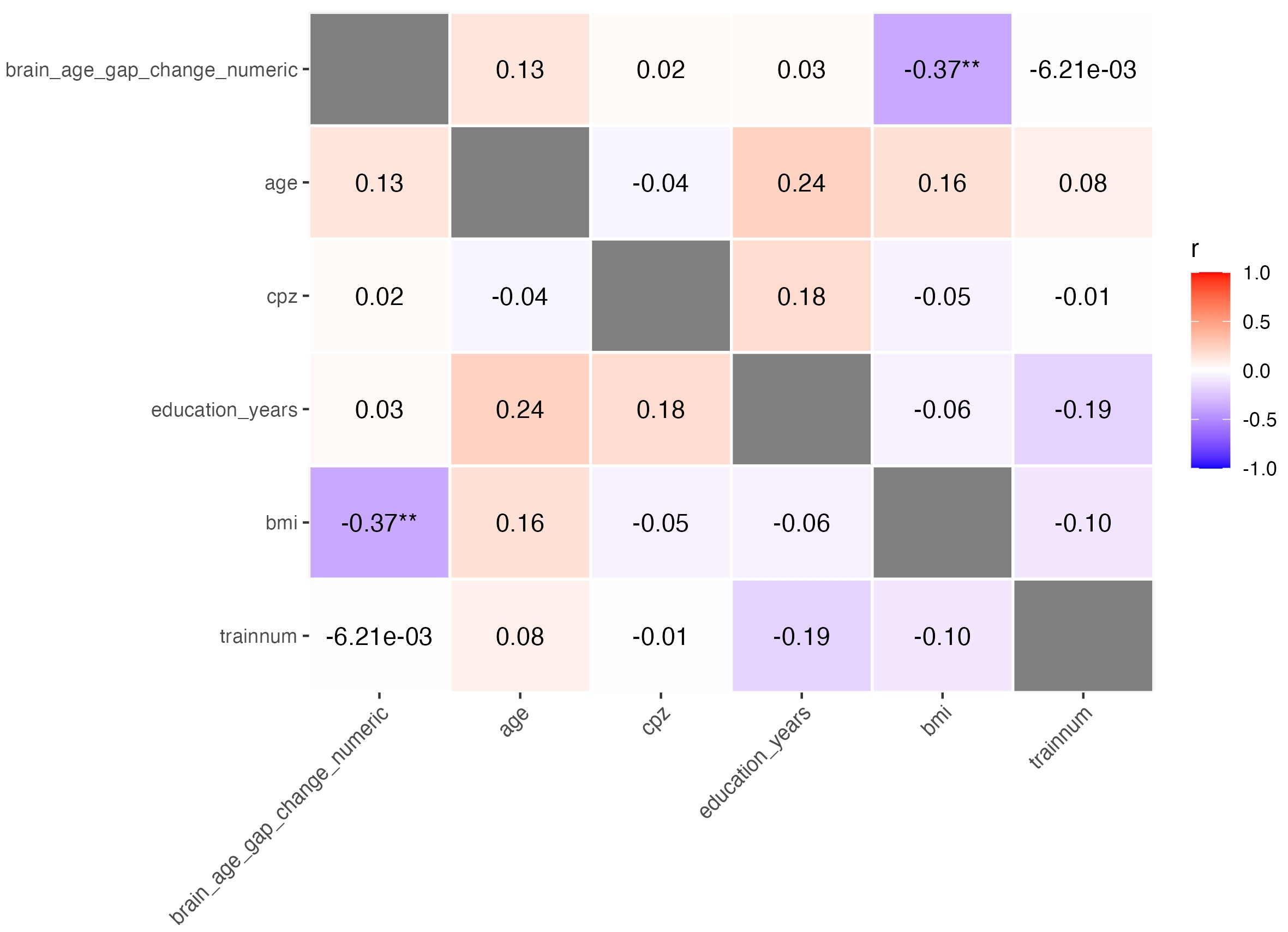

Supplement: Supplementary Figure 3 — Partial Spearman Rank Correlation Matrix of Brain Age Gap Change and Covariables. Note. brain_age_gap_change_numeric: difference in brain age gap (post-exercise - baseline); education_years: total years of formal education; trainnum: number of training sessions attended; **: p < 0.01. [file mmc3.jpg]

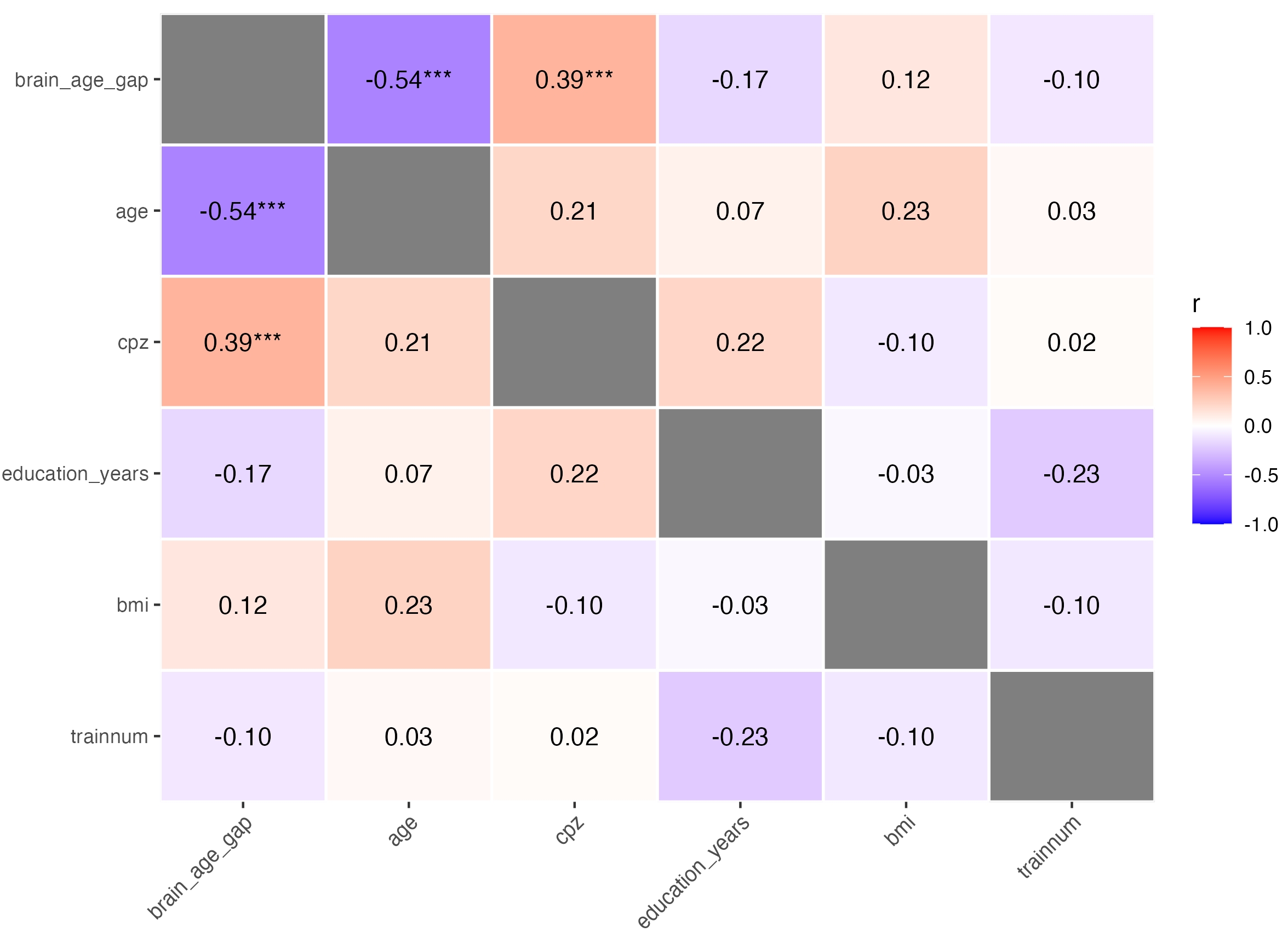

Supplement: Supplementary Figure 4 — Partial Spearman Rank Correlation Matrix of Baseline Brain Age Gap and Covariables. Note. education_years: total years of formal education; trainnum: number of training sessions attended; ***: p < 0.001. [file mmc4.jpg]

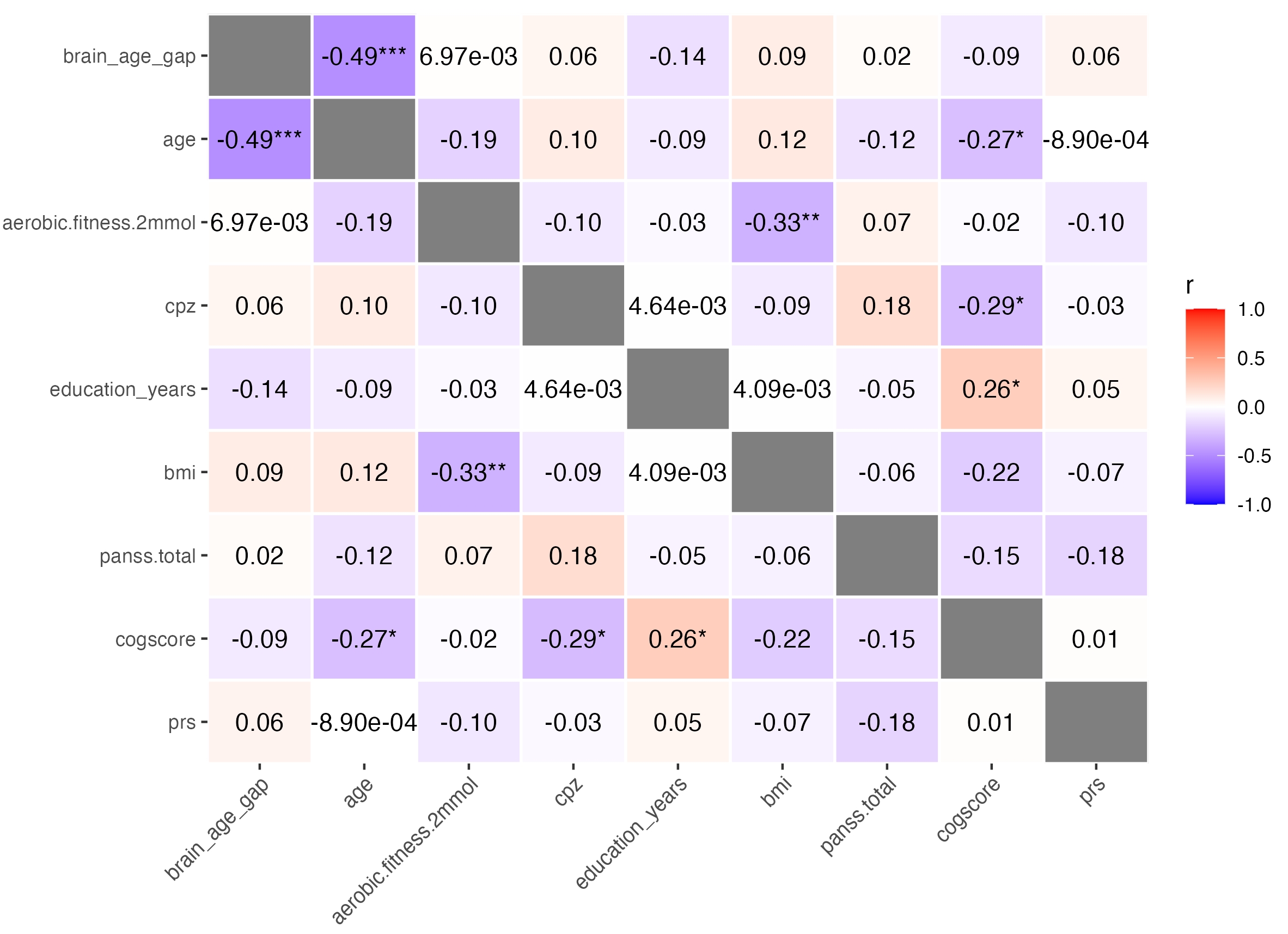

Supplement: Supplementary Figure 5 — A. Partial Correlation Between Adjusted Baseline GAF and Baseline Brain Age Gap. [file mmc5.jpg]

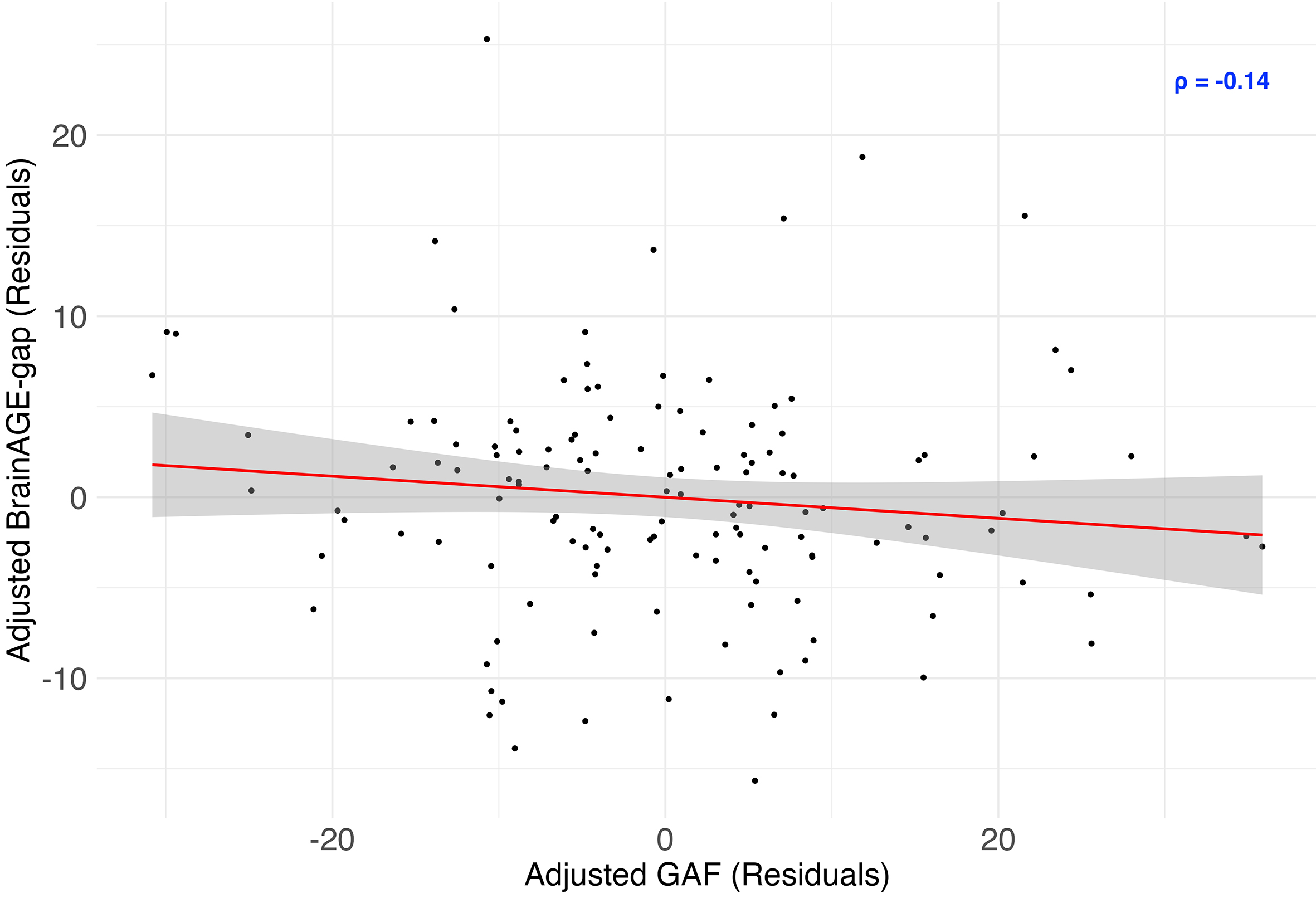

Supplement: Supplementary Figure 6 — Partial Spearman Rank Correlation Matrix of Baseline Variable. Note. Correlation coefficients are presented in scientific notation where, for example, 8.77e−03 means 8.77×10^−3^ or 0.00877. aerobic.fitness.2mmol: aerobic fitness operationalized as the exercise intensity at which blood lactate concentration reaches 2 mmol/l; education_years: total years of formal education; cogscore: composite cognitive score; *: p < 0.05; **: p < 0.01; ***: p < 0.001. [file mmc6.jpg]

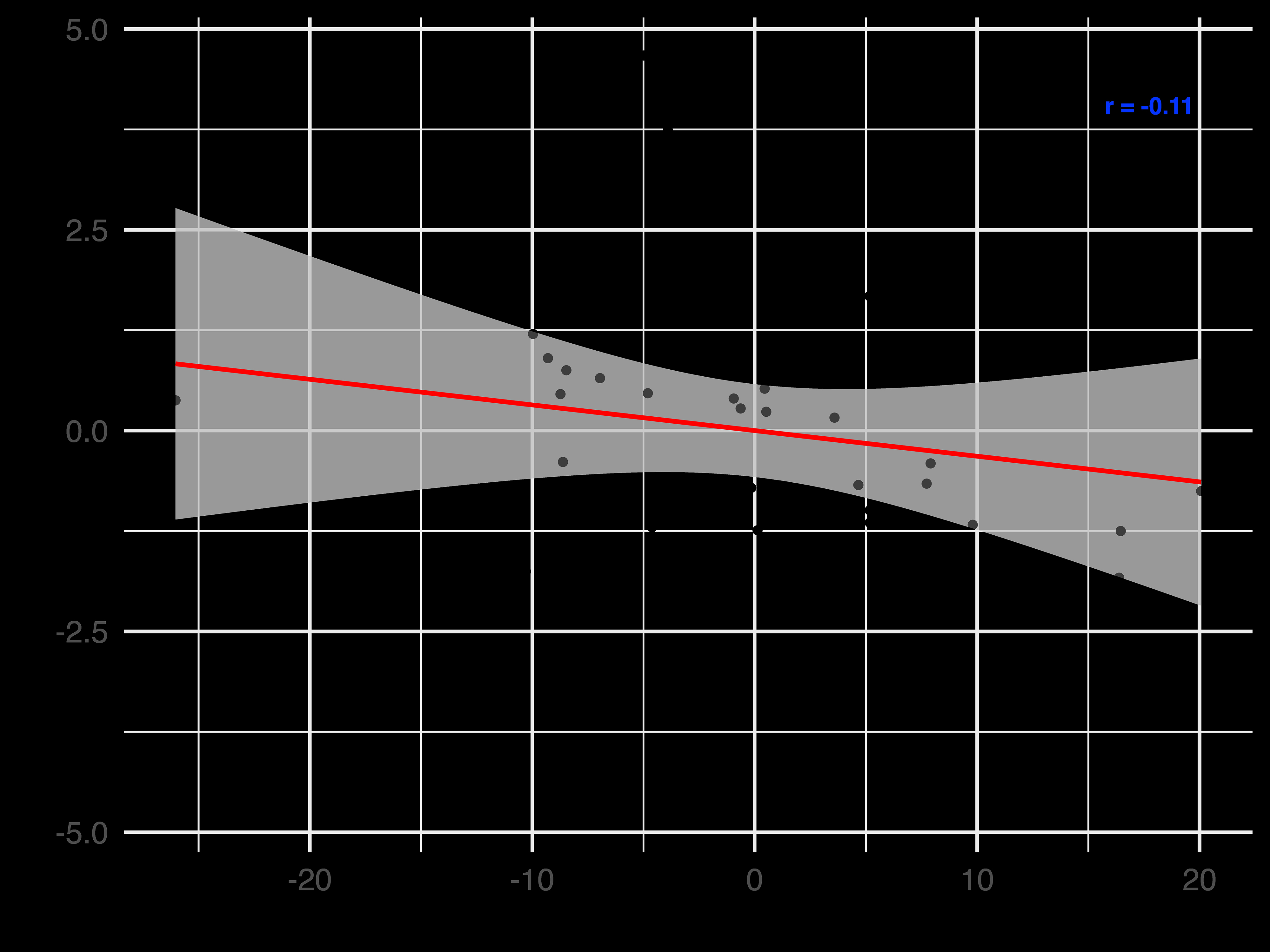

Supplement: Supplementary Figure 7 — B. Partial Correlation Between Adjusted GAF Change and Brain Age Gap Change. [file mmc7.jpg]

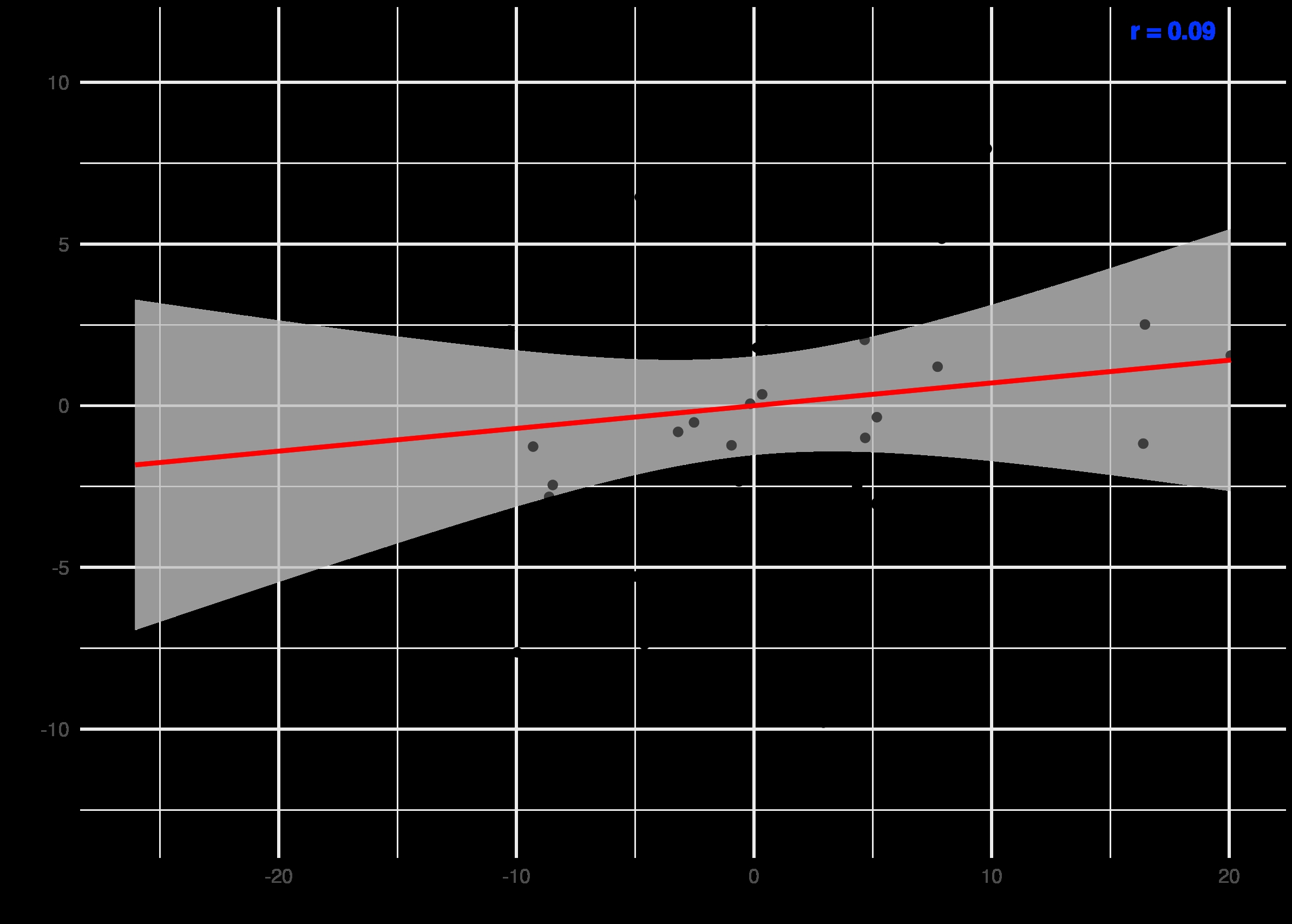

Supplement: Supplementary Figure 8 — C. Partial Correlation Between Adjusted GAF Change and Baseline Brain Age Gap. [file mmc8.jpg]

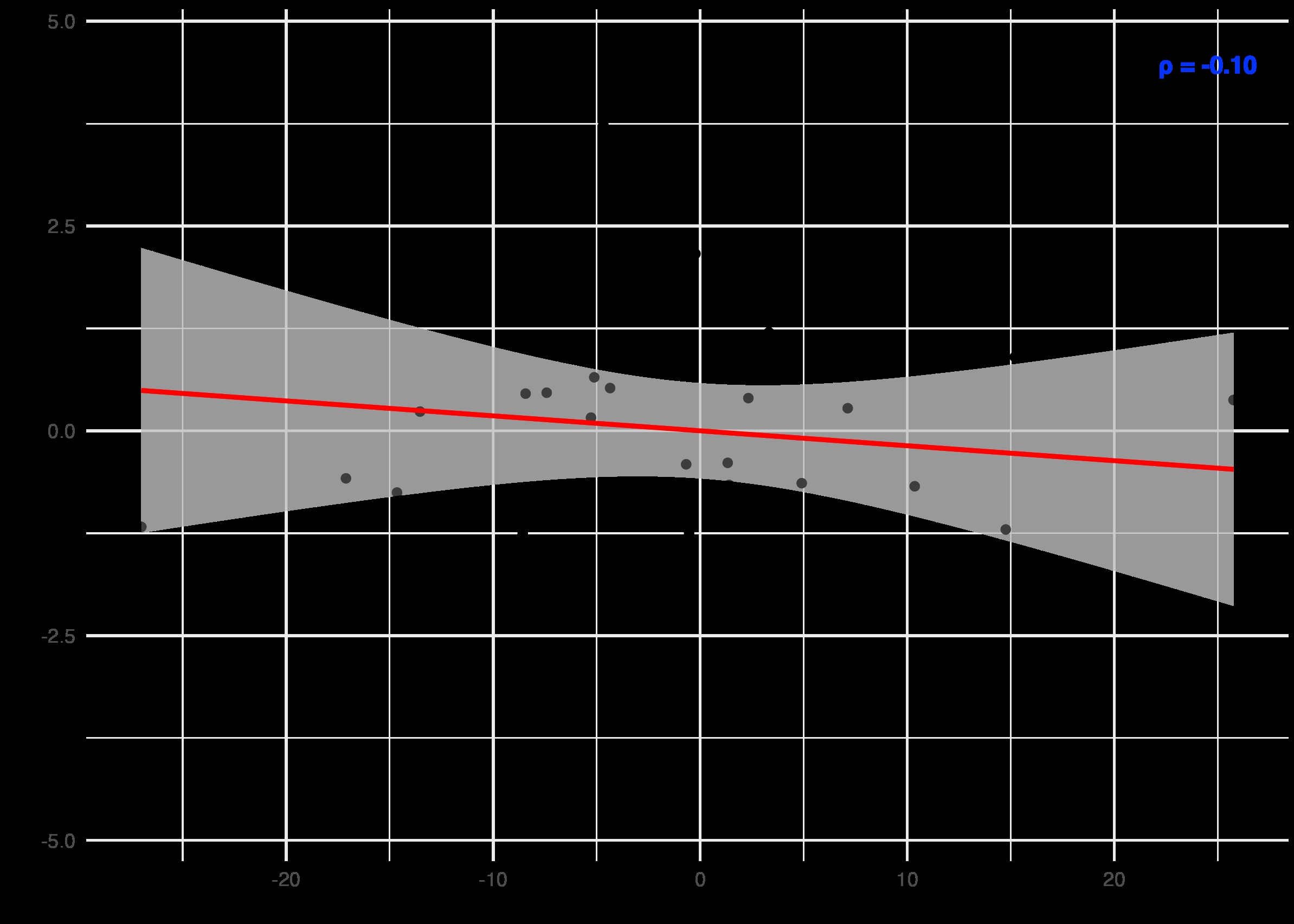

Supplement: Supplementary Figure 9 — D. Partial Correlation Between Adjusted Baseline GAF and Brain Age Gap Change. [file mmc9.jpg]

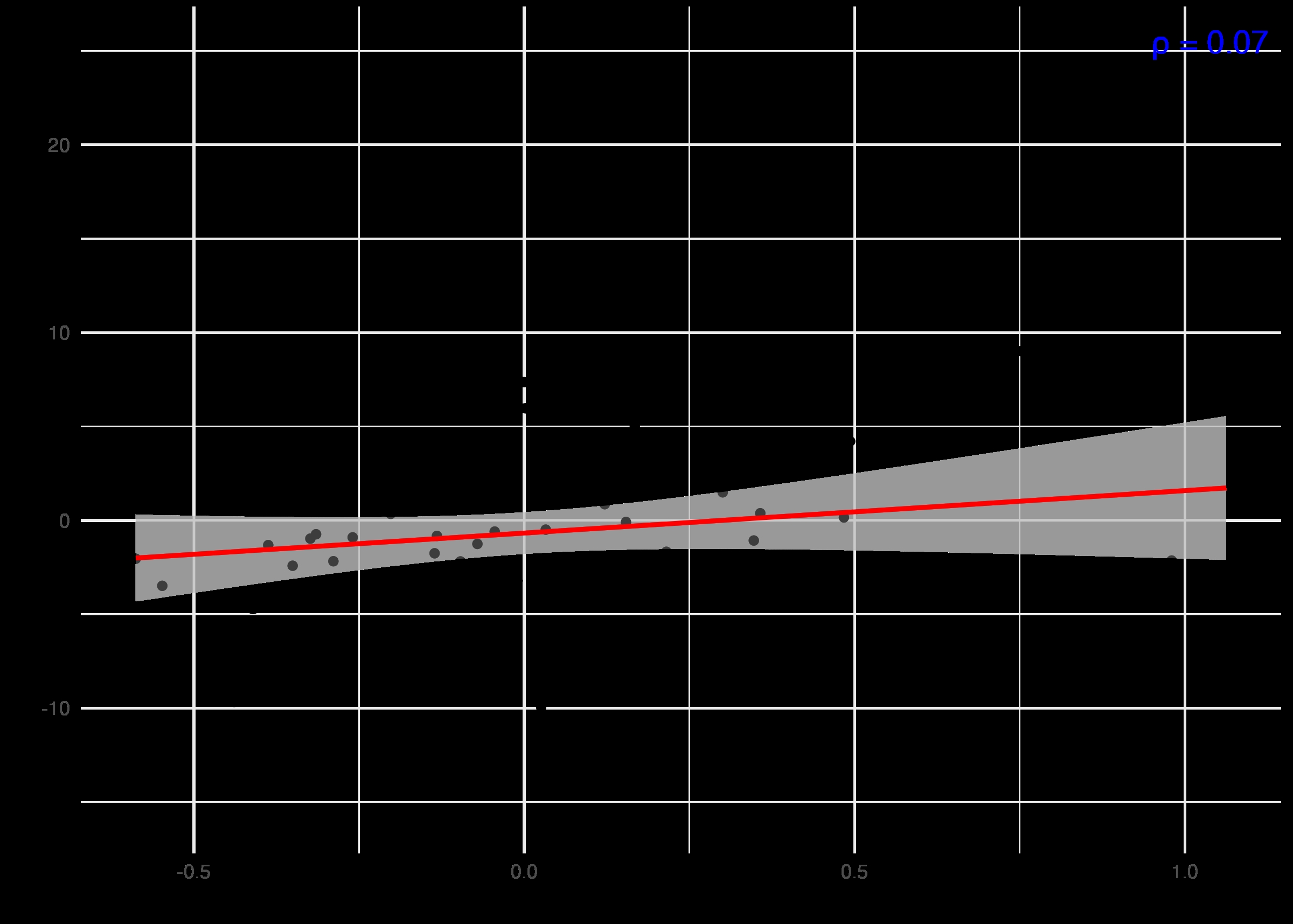

Supplement: Supplementary Figure 10 — Partial Correlation Between Adjusted Baseline Aerobic Fitness and Baseline Brain Age Gap. [file mmc10.jpg]

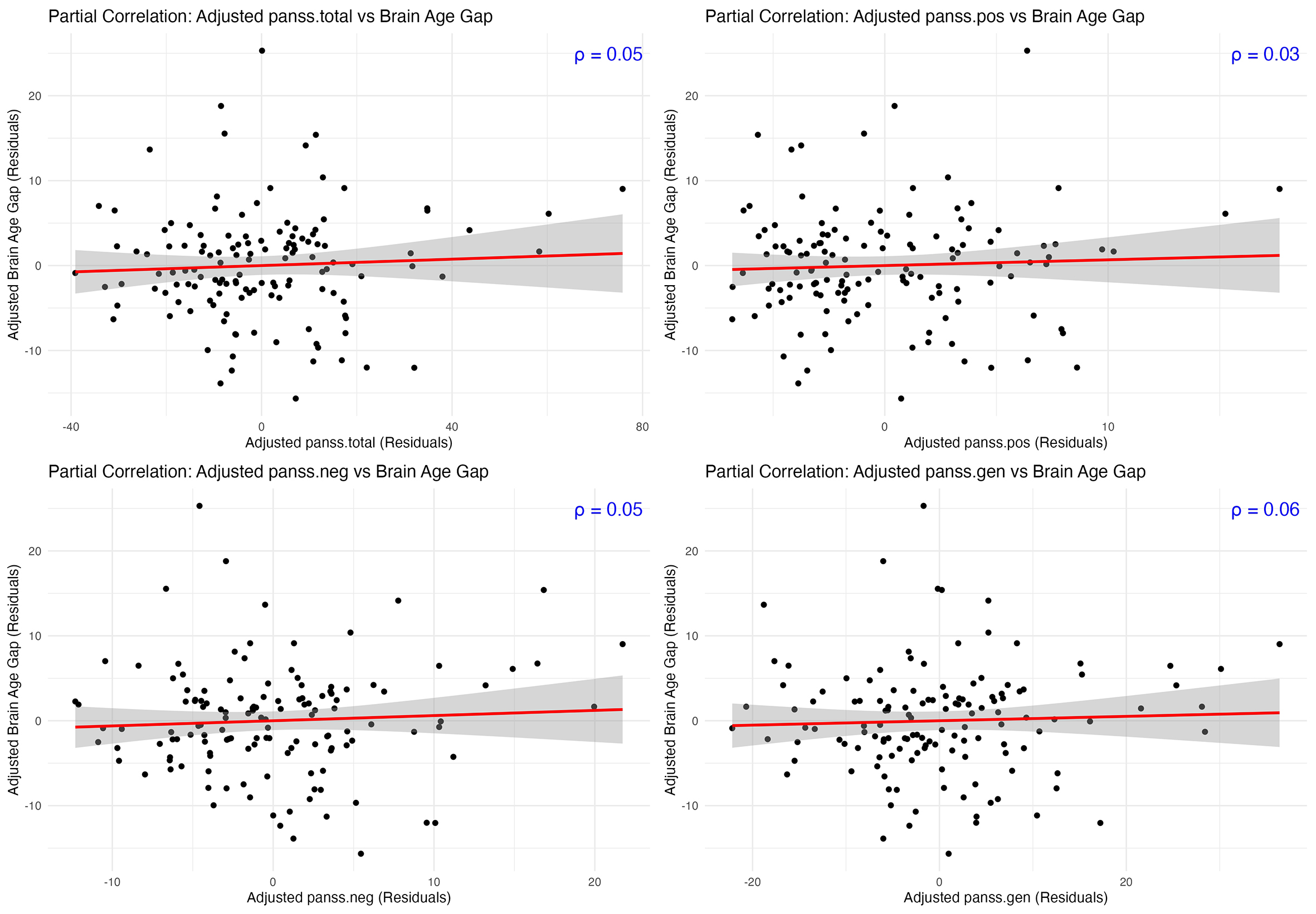

Supplement: Supplementary Figure 11 — Partial Correlation Plots: Baseline Brain Age Gap vs. Baseline PANSS Scores. Note. panss.pos: PANSS positive; panss.neg: PANSS negative; panss.gen: PANSS.general. [file mmc11.jpg]

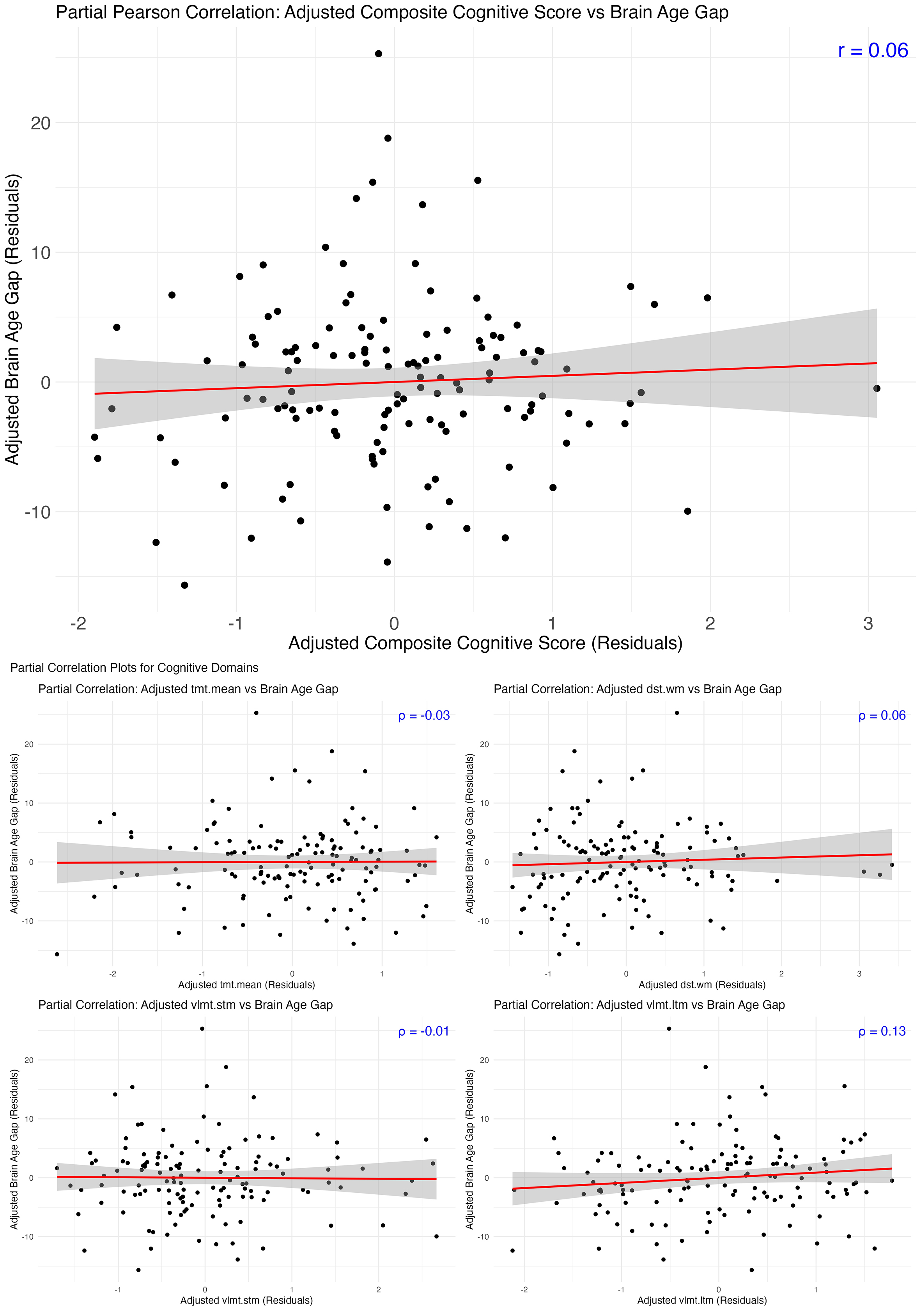

Supplement: Supplementary Figure 12 — Partial Correlation Plots: Baseline Brain Age Gap vs. Baseline Cognitive Domains. Note. tmt.mean: Trail Making Task Mean; dst.wm: Digit Span Test Working Memory; vlmt: Verbal Learning and Memory Test; stm: Short Term Memory, ltm: Long Term Memory. [file mmc12.jpg]

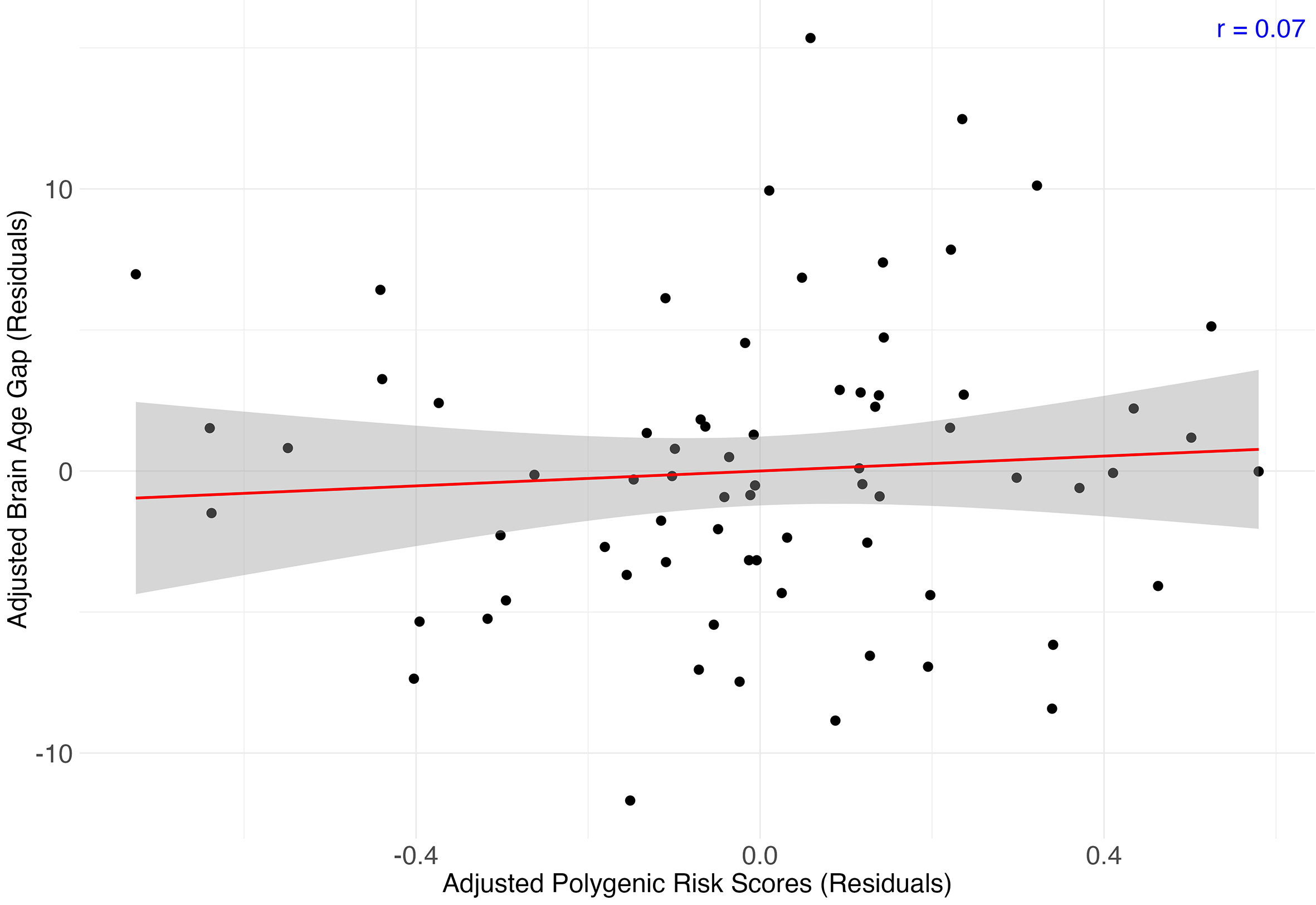

Supplement: Supplementary Figure 13 — Partial Correlation Between Adjusted Polygenic Risk Scores and Baseline Brain Age Gap [file mmc13.jpg]

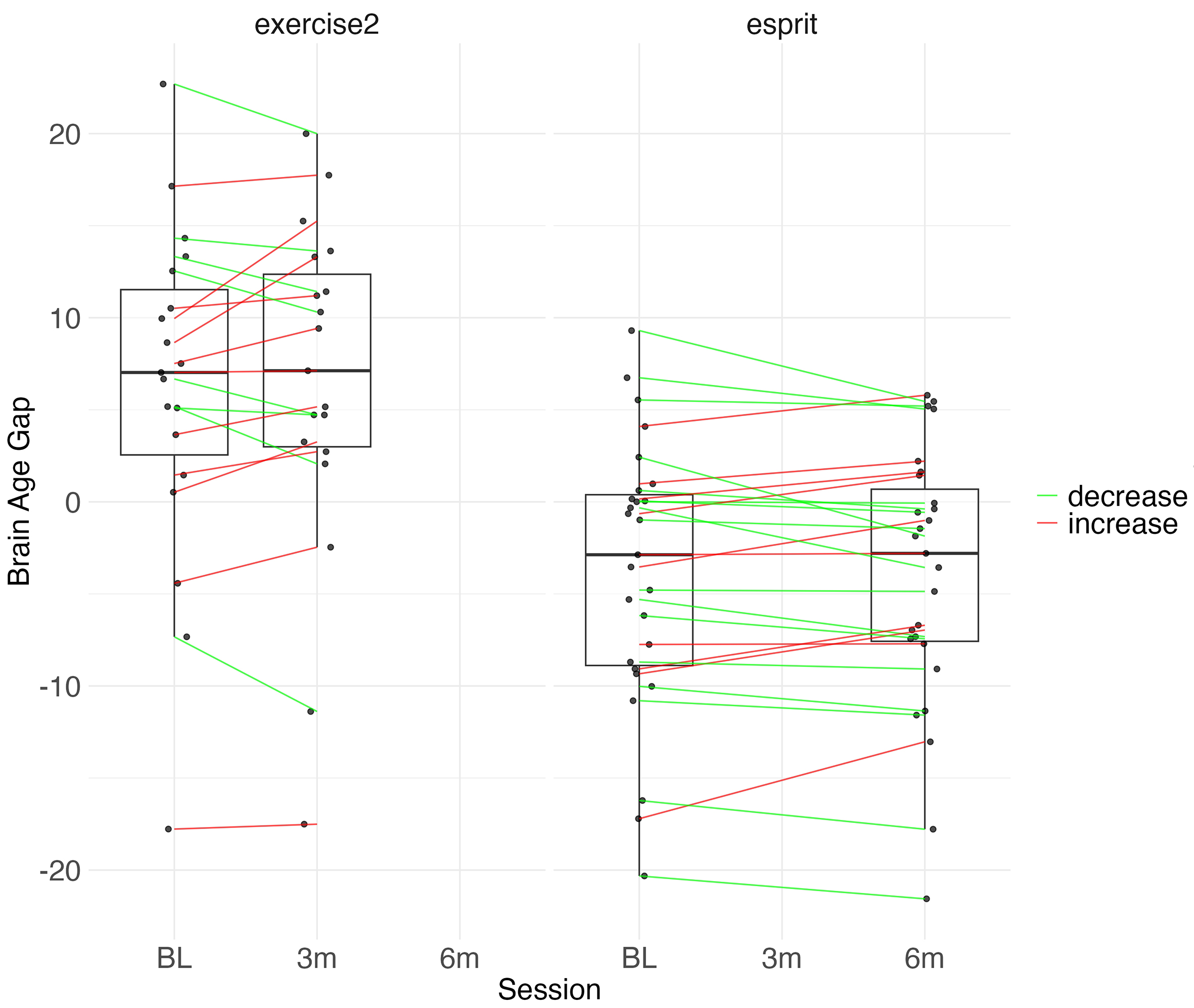

Supplement: Supplementary Figure 14 — Session Effects on Brain Age Gap by Study. Note. Boxplots represent the brain age gap at baseline and post-exercise sessions (BL: baseline; 3m: 3-month post-exercise; 6m: 6-month post-exercise). Individual participants are depicted as dots, with lines connecting them to illustrate change. Participants showing brain recovery (a decreased brain age gap) are connected to their post-exercise session in green, while those with an increased brain age gap are shown in red. The left side displays the data from the Exercise2 and the right side from ESPRIT datasets. [file mmc14.jpg]

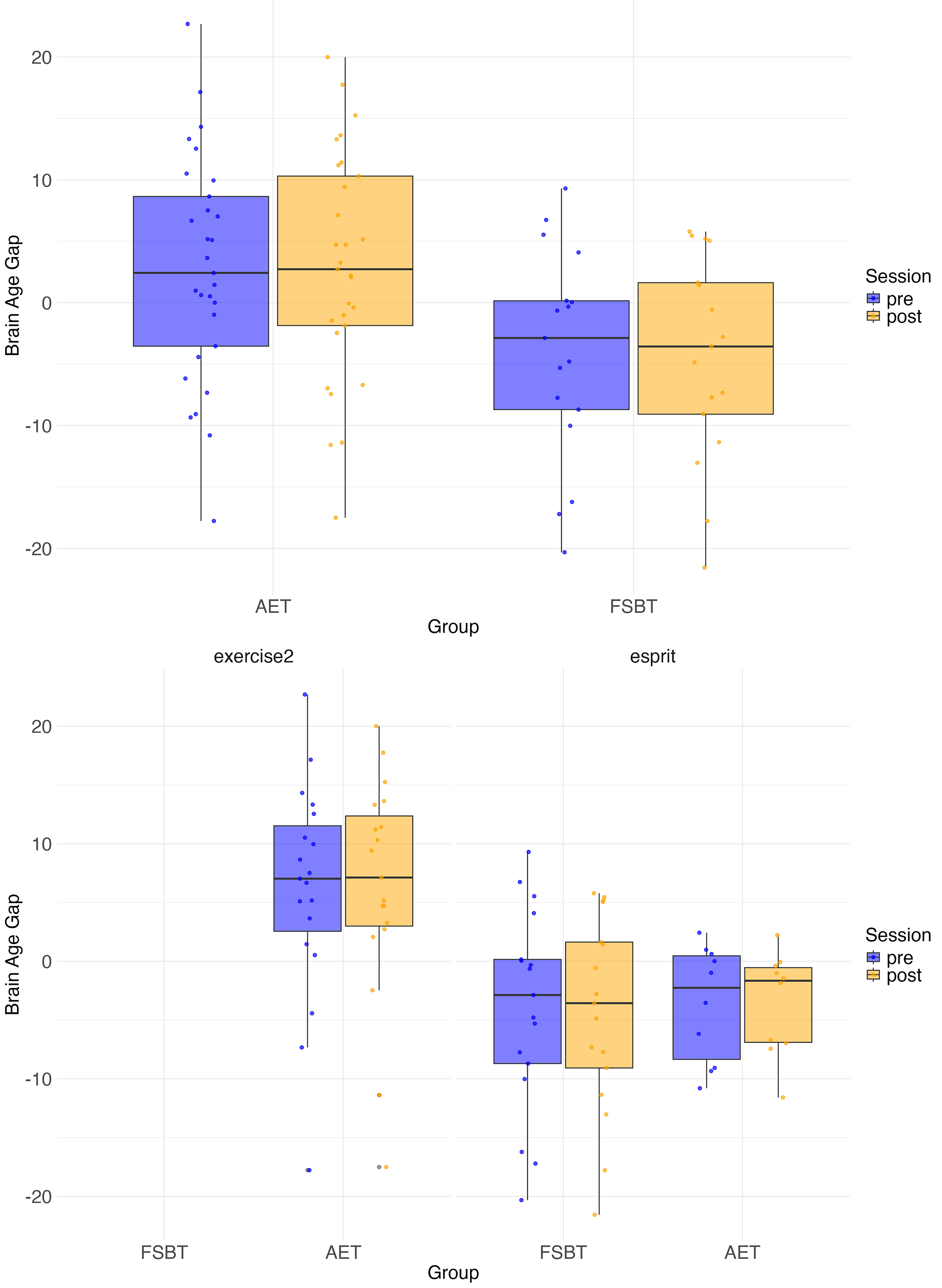

Supplement: Supplementary Figure 15 — Session Effects on Brain Age Gap by Training Group. Note. The upper graph shows brain age gaps in the pooled data pre- and post-exercise grouped by the exercise group AET (Aerobic Endurance Training) on the left side and FSBT (Flexibility-Strength-Balance Training) on the right. The lower graph displays the same information by study, Exercise2 on the left and ESPRIT on the right. Dots represent individuals. [file mmc15.jpg]

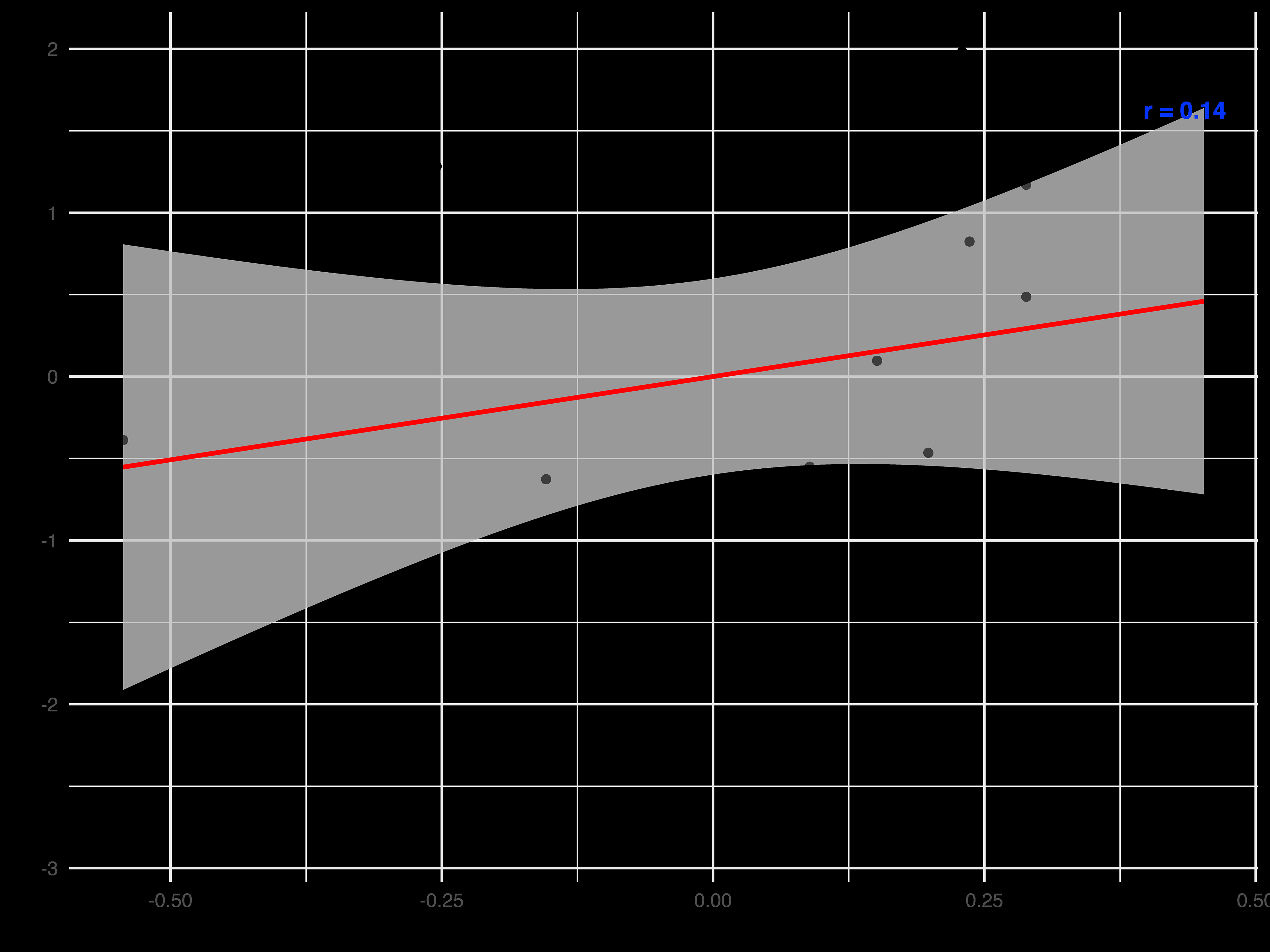

Supplement: Supplementary Figure 16 — Partial Correlation: Polygenic Risk Score vs. Post-Exercise Brain Age Gap Change. [file mmc16.jpg]

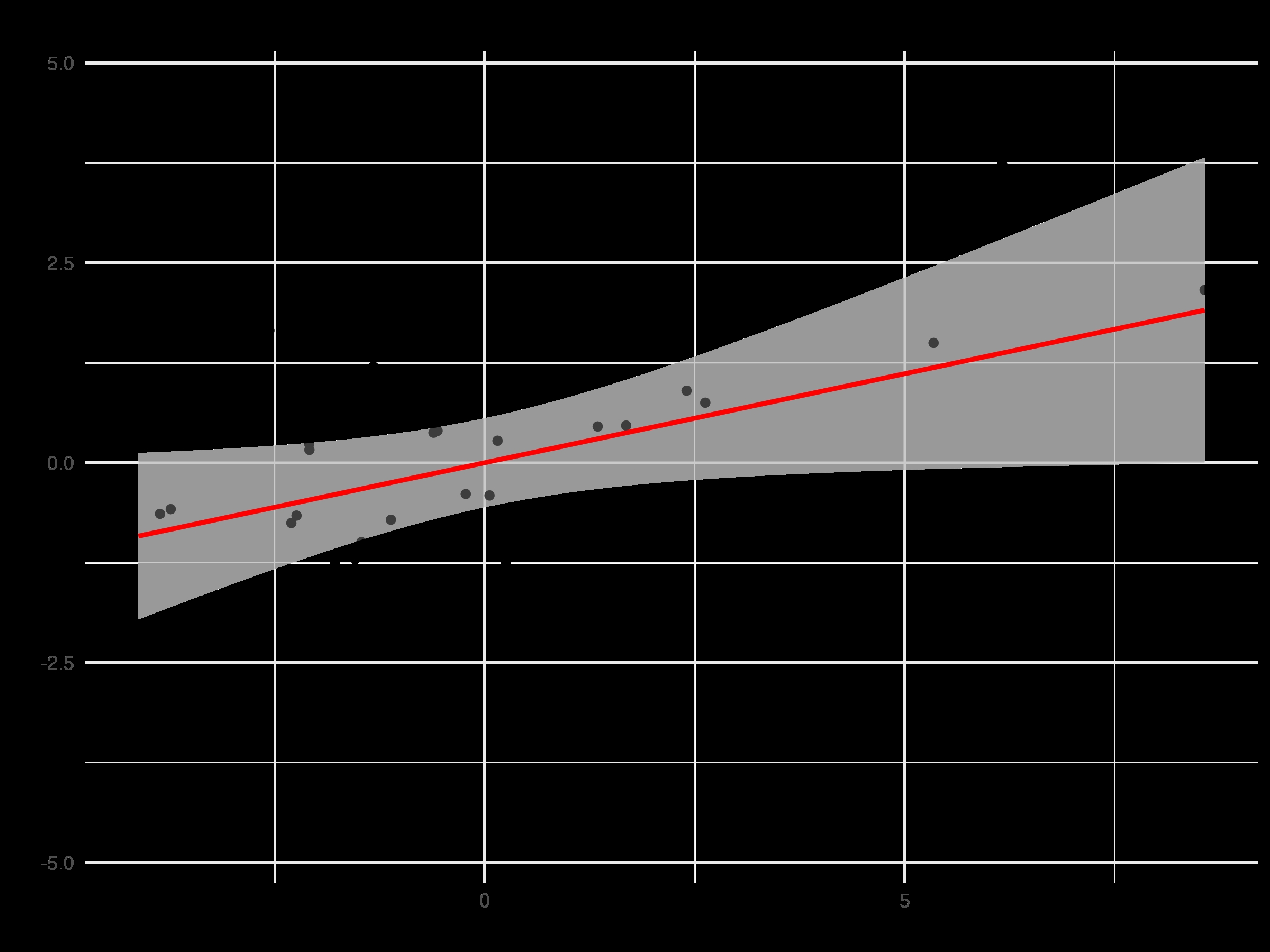

Supplement: Supplementary Figure 17 — Partial Correlation: Post-Exercise BMI Change vs. Brain Age Gap Change. [file mmc17.jpg]
